# Effect of modified Zengye decoction on age-related constipation *via* modulation of the host–microbial metabolic axis

**DOI:** 10.1093/gastro/goag031

**Published:** 2026-05-13

**Authors:** Deliang Liu, Yuying Chen, Jinzhi Pan, Zhuojun He, Yang Zhou, Guiqin Dai, Xiafei Dai, Zhiqiang Lin, Pengfei Zhao, Hongzhou Lu, Mingbin Zheng

**Affiliations:** National Clinical Research Center for Infectious Diseases, Shenzhen Third People’s Hospital, Southern University of Science and Technology, Shenzhen, Guangdong 518112, P. R. China; Institute for Hepatology, Shenzhen Third People’s Hospital, Shenzhen, Guangdong 518112, P. R. China; National Clinical Research Center for Infectious Diseases, Shenzhen Third People’s Hospital, Southern University of Science and Technology, Shenzhen, Guangdong 518112, P. R. China; The Affiliated Dongguan Songshan Lake Central Hospital, Guangdong Medical University, Dongguan, Guangdong 523808, P. R. China; National Clinical Research Center for Infectious Diseases, Shenzhen Third People’s Hospital, Southern University of Science and Technology, Shenzhen, Guangdong 518112, P. R. China; The Affiliated Dongguan Songshan Lake Central Hospital, Guangdong Medical University, Dongguan, Guangdong 523808, P. R. China; National Clinical Research Center for Infectious Diseases, Shenzhen Third People’s Hospital, Southern University of Science and Technology, Shenzhen, Guangdong 518112, P. R. China; National Clinical Research Center for Infectious Diseases, Shenzhen Third People’s Hospital, Southern University of Science and Technology, Shenzhen, Guangdong 518112, P. R. China; National Clinical Research Center for Infectious Diseases, Shenzhen Third People’s Hospital, Southern University of Science and Technology, Shenzhen, Guangdong 518112, P. R. China; National Clinical Research Center for Infectious Diseases, Shenzhen Third People’s Hospital, Southern University of Science and Technology, Shenzhen, Guangdong 518112, P. R. China; National Clinical Research Center for Infectious Diseases, Shenzhen Third People’s Hospital, Southern University of Science and Technology, Shenzhen, Guangdong 518112, P. R. China; Institute for Hepatology, Shenzhen Third People’s Hospital, Shenzhen, Guangdong 518112, P. R. China; Guangdong Key Lab for Diagnosis & Treatment of Emerging Infectious Diseases, Shenzhen Third People’s Hospital, Shenzhen, Guangdong 518112, P. R. China; National Clinical Research Center for Infectious Diseases, Shenzhen Third People’s Hospital, Southern University of Science and Technology, Shenzhen, Guangdong 518112, P. R. China; The Affiliated Dongguan Songshan Lake Central Hospital, Guangdong Medical University, Dongguan, Guangdong 523808, P. R. China

**Keywords:** age-related constipation, gut microbiota, metabolomics, Chinese herbs, short-chain fatty acids

## Abstract

**Background:**

Constipation is a common digestive disorder in the elderly caused by weakened intestinal peristalsis and reduced mucus secretion that significantly impacts quality of life. Current treatments typically provide only temporary symptomatic relief and may lead to dependence and adverse effects.

**Methods:**

This study investigated the therapeutic effects of a modified traditional Chinese formula, modified Zengye decoction (MZD) on age-related constipation by modulating the gut microbiome and metabolomics. Aged constipated rats were gavage-fed with or without MZD. After detection of the indicators related to the disease, the microbial and metabolic profiles were generated for all the fecal samples by using 16S rRNA gene sequencing and ^1^H nuclear magnetic resonance (^1^H NMR) spectroscopy, respectively.

**Results:**

MZD effectively alleviated constipation symptoms in aged mice by enhancing the intestinal peristalsis and antioxidant capacity. Gut microbiome analysis revealed that MZD significantly altered the abundance of Firmicutes, Bacteroidetes, and Actinobacteria. Specifically, the abundance of beneficial bacteria such as *Corynebacterium*, *Roseburia*, and *Clostridium* increased by 6-fold, 4-fold, and 3-fold, respectively. These changes in microbial composition enhanced the production of short-chain fatty acids (SCFAs), including acetate, propionate, and butyrate. Additionally, MZD significantly increased the expression of Mucin 2 protein and the moisture content of the small intestine, while decreasing pro-inflammatory cytokines such as tumor necrosis factor-alpha, interleukin-1 beta, and interleukin-6, and increasing anti-inflammatory interleukin-10, which may be attributed to the elevated levels of SCFAs.

**Conclusions:**

By effectively regulating the gut microbiome and SCFA metabolism, MZD demonstrated significant anti-inflammatory and mucus-secretion-promoting effects, showing therapeutic potential for age-related constipation, enteritis, and other inflammation-related intestinal diseases.

## Introduction

Constipation is a prevalent gastrointestinal complaint that crosses all ages, sexes, and races, with a global lifetime incidence approaching 79% [1]. Beyond the age of 65 years, the disorder becomes markedly more common and is no longer a mere nuisance [2]. Epidemiological data now link chronic constipation to heightened risks of colorectal cancer, cardiovascular events, and even dementia, presumably because retained fecal matter amplifies systemic exposure to pro-inflammatory bacterial metabolites [3]. Paradoxically, the very drugs prescribed to alleviate the problem—osmotic or stimulant laxatives—often perpetuate it: tolerance develops, electrolyte disturbances emerge, dehydration worsens, and nutrient absorption is impaired, making long-term use in frail patients problematic [4]. In traditional Chinese medicine, bowel motility has for a long time been approached from a different vantage point, emphasizing the restoration of endogenous secretion and motility rather than episodic purgation. One exemplar prescription is Zengye decoction, first recorded in the 1798 Qing-dynasty text Wen Bing Tiao Bian. The original formula, composed of *Scrophularia ningpoensis*, *Radix rehmanniae*, and *Ophiopogon japonicus* in at 5:4:4 weight ratio, was designed to generate fluid and moisten dryness, and it remains a front-line therapy for Sjögren’s syndrome and constipation in China [5, 6]. Over the past decade, Zengye decoction has also shown efficacy in insulin-resistant diabetes and acute pancreatitis complicated by acute kidney injury [7–9], while our own work demonstrated that it restores bowel function in aged rats by rebalancing the gut microbiota and amino-acid metabolome [10].

However, age-related constipation is characterized by diminished mucosal lubrication, attenuated colonic motility, and persistent dysbiosis—changes that demand an even broader, multi-target approach. Thus, we modified Zengye decoction to create modified Zengye decoction (MZD). In this new herbal matrix, *S. ningpoensis* (Xuanshen) and *O. japonicus* (Maidong) continue to rehydrate the colon and replenish mucus; *Astragalus membranaceus* (Huangqi) was added to enhance smooth-muscle contraction and enteric neural tone; low-dose *Semen Raphani* (Laifuzi) and *Folium Sennae* (Fanxieye) provided gentle, nonexplosive peristaltic drive; and the prebiotic stachyose selectively nourished beneficial microbiota, amplifying microbial metabolite feedback to the host.

In this study, we examined the potential mechanisms of MZD in rat models of age-related constipation through metabolomics and intestinal microbiome analyses, focusing on the host–microbial metabolic axis to clarify how the gut microbiota and its metabolites interact with the host to influence age-related constipation. By integrating these data, we aimed to evaluate the therapeutic potential of MZD for age-related constipation, enteritis, and other inflammation-related intestinal diseases.

## Materials and methods

### Preparation of MZD

The MZD preparation was carried out as follows: water was added to a mixture of 20 g of *S. ningpoensis*, 20 g of *O. japonicus*, 10 g of *A. membranaceus*, 5 g of *Semen Raphani*, 5 g of *Folium Sennae*, and 20 g of stachyose at a ratio of 10:1 (water:plant material, v/w). The mixture was boiled for 2 h and filtered, and the dregs were re-boiled for an additional 2 h after the addition of freshwater at a ratio of 10:1 (water:plant material, v/w). After filtering, the supernatant was collected, concentrated, and lyophilized to freeze-dried powder. Afterward, the powder was reconstituted with distilled water at 0.13 g/mL (MZD low-dose group, MZD-L) and 0.39 g/mL (MZD high-dose group, MZD-H) and the MZD group of experimental animals treated through intragastric administration (0.01 mL/g body weight).

### Quality control of MZD by using high-pressure liquid chromatography

Quality control of the MZD was carried out by using high-pressure liquid chromatography (HPLC) to determine the cinnamic acid content in the MZD. Analytical-grade cinnamic acid (≥98%, Sigma-Aldrich, St Louis, USA) was accurately weighed and dissolved in 50% (v/v) methanol to obtain a stock solution of 1 mg/mL. Serial working standards (1, 5, 10, 50, and 100 µg/mL) were prepared by diluting the stock with 50% methanol as indicated in the calibration table. Test samples were diluted 10-fold with 50% methanol prior to injection. Chromatographic separation was performed on a Waters Arc HPLC system fitted with a ZORBAX SB-C18 column (4.6 × 250 mm, 5 µm) thermostatted at 30°C. The mobile phase consisted of 0.1% phosphoric acid in water and acetonitrile (50:50, v/v) delivered isocratically at 1.0 mL/min over 10 min. The injection volume was 10 µL and ultraviolet (UV) detection was carried out at 268 nm. Under these conditions, cinnamic acid eluted as a single symmetrical peak with acceptable resolution and no interfering matrix peaks.

### HPLC fingerprint analysis

The chromatographic conditions used for fingerprint analysis were optimized based on those mentioned for quantitative analysis. The mobile phase consisted of 0.1% phosphoric acid (Solvent A) and acetonitrile (Solvent B) at a flow rate of 1.0 mL/min, which followed a gradient program of 0–25 min, 5%–17% B; 25–50 min, 17%–25% B; 50–80 min, 25%–55% B; 80–95 min, 55%–95%. For similarity analysis, based on the fingerprint chromatographic conditions for the fingerprint method, all the data were entered into the Similarity Evaluation System for Chromatographic Fingerprint of Traditional Chinese Medicines (2012 version) to obtain common peaks with a peak area of >0.09% of the total peak area during the match. This calculated and generated simulated reference fingerprints while separately counting the correlation coefficients of similarity between nine batches of MZD and simulated reference fingerprints.

### Evaluation of MZD treatment for constipation therapy *in vivo*

A total of 40 male-specific pathogen-free Sprague-Dawley rats (18–20 weeks old, 280–300 g) were purchased from the Laboratory Animal Centre of Guangdong (Guangzhou, China; Permission No: SCXK (Yue) 2013–0002). The rats were housed in a sterile laminar flow environment at the SPF Experimental Animal Center of Guangzhou University of Chinese Medicine under standard conditions (12/12 h light–dark cycle at 24°C, 50%–70% humidity). The animals were individually housed and fed standard chow. They were randomly assigned to the health group (*n *= 10), untreated group (*n *= 10), MZD-L (*n *= 10), or MZD-H (*n *= 10). An age-related constipation model was established in the untreated, MZD-L, and MZD-H groups through multiple stimulations over 4 weeks, as previously described [11]. The MZD-L and MZD-H groups received 1.3 and 3.9 g/kg of MZD, respectively, by gavage for 5 weeks, while the healthy and untreated groups received an equivalent volume of sterile saline [7]. The defecation frequency and stool characteristics were recorded weekly. At the end of Week 9, the rats were euthanized by using CO_2_ and subjected to necropsy. The animal study protocol was approved by the Ethics Committee of Guangzhou University of Chinese Medicine. All procedures of animal treatment were in accordance with the National Guidelines for Experimental Animal Welfare (Ministry of Science and Technology, China, 2006) and the United States National Institutes of Health Guide for Care and Use of Laboratory Animals at the Centre for Animal Experiments, making significant efforts to minimize both suffering and the number of animals used.

### Sample collection and pathological examination

Fecal samples were collected from individual animals on Day 63 (from 8 a.m. to 4 p.m.) and rapidly frozen and kept in liquid nitrogen to be used for gene sequencing. Blood samples were collected and centrifuged at 3,500 rpm at 4°C for 15 min. The plasma samples were stored at −80°C until the time of analysis. The ink-propelling rate in the small intestine was measured 30 minutes after the intragastric administration of ink. The moisture contents in the small intestine were calculated as follows: moisture (%) = [(wet weight − dry weight)/wet weight] × 100%.

The colonic tissue was removed, washed with physiological saline, and fixed with 10% neutral buffer formalin for histopathology analysis. After fixation, the colonic tissues were embedded in paraffin, cut into 10-mm slices, and stained with hematoxylin–eosin stain. Images were obtained and studied under a biological microscope (Leica DM5000 B, Germany).

### Biochemistry assays

The biochemical indexes of plasma superoxide dismutase (SOD) and malondialdehyde (MDA) were measured according to the instructions for the enzymatic kits (Nanjing JianCheng Bioengineering Institute, Nanjing, China). The optical density value was detected by using ultraviolet spectrophotometry (Shimadzu UV-2450, Japan). Cytokines in the colon were measured by using enzyme-linked immunosorbent assay (ELISA) according to the manufacturer’s instructions.

### Western blot analysis

Colon proteins were extracted according to tissue protein-extraction protocols. The protein concentrations were determined by using the Protein Assay Kit and adjusted to a known concentration before electrophoresis. The rabbit monoclonal anti-MUC2 antibody (Abcam, ab272692, Cambridge, UK) was incubated overnight at 4°C in antibody diluent, followed by 2 h of incubation at room temperature with goat anti-rabbit secondary antibody (Abcam, ab205718, Cambridge, UK). The chemiluminescence of the immunoreactive protein bands was captured by using a chemiluminescence imager and signal intensities were quantified by using ImageJ 1.80 software. The protein expression was normalized to β-actin.

### RNA extraction and quantitative real-time PCR analysis

Colon RNA was extracted according to previous literature [12]. The RNA concentration was determined by using a NanoDrop ND-1000 (NanoDrop Technologies, USA) and reverse transcription was performed by using a reverse transcriptase kit (Toyobo, Osaka, Japan) according to the manufacturer’s instructions. Real-time PCR was performed by using a SyBr Green PCR system (SYBR^®^ Green Real-time PCR Master Mix; Toyobo, Osaka, Japan). The primer sequences were described in Supplementary Table S2. Glyceraldehyde-3-phosphate dehydrogenase (GAPDH) was amplified as an internal reference and the relative expression of mRNA to GAPDH was calculated by using the 2^−△△^Ct method.

### 16S rRNA gene microbiota profiling

The 16S rRNA V4 region was amplified and sequenced by using the Illumina HiSeq 2500 PE250 platform (New England Biolabs, USA). The primer sequences were 515 F 5′-GTGCCAGCMGCCGCGGTAA-3′ and 806R 5′-GGACTACHVGGGTWTCTAAT-3′, and the reverse primer contained unique barcode sequences and appropriate adapter tags for each PCR product to distinguish different samples. Each 50 μL of the PCR mixture consisted of 25 μL of PCR master mix (NEB Phusion High-Fidelity PCR Master Mix), 4 μL of PCR primer cocktail (515F-806R), 30 ng of DNA template, and an appropriate volume of nuclease-free as needed. The PCR reaction occurred as follows: 3 min at 98°C; 30 cycles of 45 s at 98°C, 45 s at 55°C, and 45 s at 72°C; followed by 10 min of final elongation at 72°C. The PCR products were detected by using 1% agarose gel electrophoresis and purified with AmpureXPbeads (AGENCOURT) to remove nonspecific products.

The 16S microbial data were analysed by using QIIME version 1.9. Similar sequences (97%) were combined into operational taxonomic units (OTUs) by using an open-reference OTU-picking strategy and representative sequences for each OTU were aligned by using the Python Nearest Alignment Space Termination method [13]. Diversity was calculated by using QIIME software and visualization was performed in R software (Version 2.15.3). All raw data have been deposited at the National Center for Biotechnology Information database (under the BioProject accession number PRJNA415319).

### Fecal metabolome analysis

Fecal samples were extracted by using the optimized method described by Wu *et al*. with slight modifications [14]. The 1D ^1^H NMR spectra were recorded at 298 K on a Bruker AVIII 600-MHz spectrometer (Brulcer Biospin, Germany) at 600.13 MHz. The parameters used were in accordance with the method described by Dong *et al*. [15]. A range of 2D NMR experiments was used to assign the NMR signal as previously reported [16]. TOPSPIN (V2.1, Bruker Biospin, Germany) was used to manually correct the phase and baseline distortion of all ^1^H NMR spectra of the fecal samples and to calibrate the sodium 3-(trimethylsilyl)propionate-d_4_ (TSP) (δ0.00). The metabolites were simultaneously identified based on the Human Metabolome Database (http://www.hmdb.ca/) and metabonomics toolbox (Chenomx NMRSuit 7.6, Chenomx, Canada) as well as published work [17].

### Statistical analysis

All values were expressed as mean ± SD. Multivariate statistical analysis was performed by using SIMCA-P 13.0 (Umetric, Umeå, Sweden) to process the acquired NMR data. Statistical analyses (Student’s *t*-tests) were performed as two-sample *t*-tests. Differences between more than two groups with only one variable were assessed by using one-way analysis of variance (ANOVA) and the Bonferroni post hoc test. Significant *P* values associated with microbial clades identified by using the linear discriminant analysis effect size (LEfSe) were corrected for multiple-hypothesis testing by using the Benjamini–Hochberg false discovery rate (FDR) method.

## Results

### Quality control of MZD

**Figure 1:**
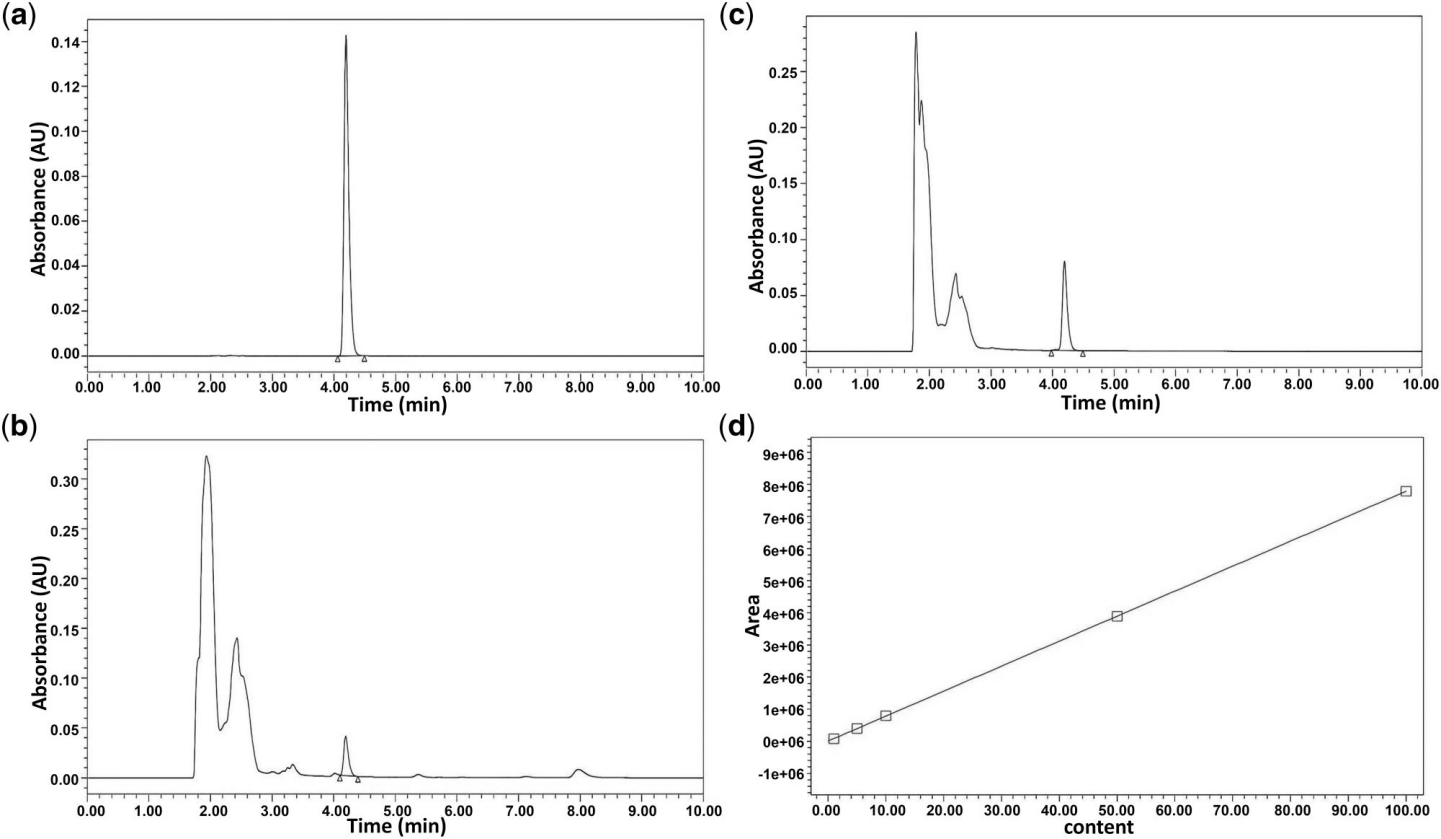
HPLC chromatogram of cinnamic acid. (a–c) Representative HPLC chromatogram of cinnamic acid in reference substance, *Scrophularia ningpoensis* decoction, and MZD. (d) Standard curves of cinnamic acid.

The HPLC chromatograms of the reference substance and cinnamic acid in both *S. ningpoensis* decoction and MZD are presented in Fig. 1a–c. The results showed that both *Scrophularia ningpoensis* decoction and MZD contain cinnamic acid (retention time: 4.19 min). Cinnamic acid contents were calculated by using the standard curve (Fig. 1d) and the concentrations were 0.26 mg/mL for MZD-L and 0.74 mg/mL for MZD-H. The optimized chromatographic conditions resulted in good separation of the constituents in all samples. Fingerprint analysis was established based on the HPLC data of nine batches of MZD (S1–S9). All data were entered into the Similarity Evaluation System for Chromatographic Fingerprint of Traditional Chinese Medicines (2012 version) to obtain common peaks with a peak area of >0.09% of the total peak area during the match. A total of 13 common peaks (Peaks 1–13) were found in these chromatograms along with a reference fingerprint (R) spectrum (Supplementary Figure S1). The similarity values of the HPLC fingerprints were matched by calculating the correlative coefficients between the MZD samples and the reference fingerprint (R). The similarities were evaluated with an overall value of >0.990, indicating good batch-to-batch similarity (Supplementary Table S1).

### MZD relieved disease symptoms of aged constipated rats

**Figure 2:**
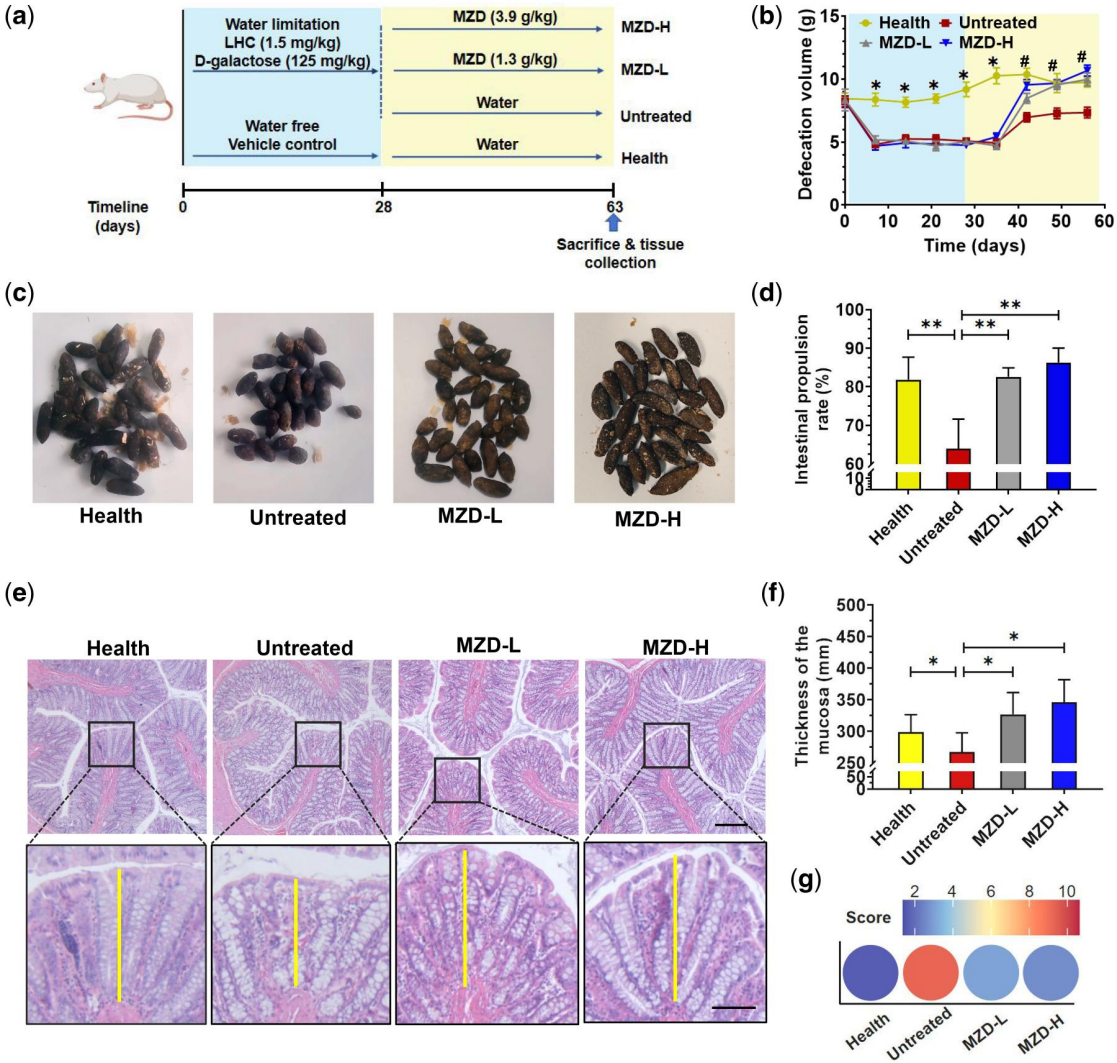
MZD attenuated the constipation symptoms in aged rats. (a) Schematic summary of animal experiment. (b) Change in defecation volume over time with different treatments. Values are expressed as mean ± SD, Student’s *t*-test *P* < 0.05, significant difference, *P < 0.05, compared with the healthy group. ^#^*P *< 0.05, compared with untreated group. (c) Effects of MZD on fecal traits. (d) Plot of the ink-propelling rate of the small intestine. (e) Histological examination of colonic mucosa. Vertical line indicates the thickness of the colon mucosa. Upper scale bars, 250 µm; bottom scale bars, 100 µm. (f) Thickness of the colon mucosa. (g) Histopathological scoring of colonic sections. Values are expressed as mean ± SD. ANOVA *P* < 0.05, significant difference, *P < 0.05, **P < 0.01.

To investigate the protective effect of MZD on aged constipated rats, an aged constipated rat model was established by using water limitation, the intragastric administration of loperamide hydrochloride (LHC), and the subcutaneous injection of D-galactose. Subsequently, MZD was administered to the rats via gavage. The specific experimental process is shown in Fig. 2a. In brief, aged constipated rats were induced by daily administration of D-galactose (125 mg/kg) and LHC (1.5 mg/kg) under water limitation for 28 days, followed by treatment with high-dose (3.9 g/kg) or low-dose (1.3 g/kg) MZD for 35 days until sacrifice at Day 63. Compared with healthy rats, untreated constipated rats exhibited a significant reduction in the defecation volume (DV, a direct index of constipation) after modeling, with feces that were drier, harder, and shorter. In contrast, the MZD-treated group showed a rapid recovery to a normal DV after modeling, with feces that were more moist, softer, and longer, particularly in the MZD-H group (Fig. 2b and c). The results indicated that MZD treatment improved the intestinal condition of aged constipated rats, as confirmed by a higher intestinal propulsion rate (Fig. 2d). The untreated group exhibited lower serum SOD levels and higher MDA levels than the MZD-treated group on Day 63, indicating that the MZD had alleviated oxygen free radical injury *in vivo* (Supplementary Fig. S2). Intestinal pathological results showed that the untreated group had mild lymphocytic infiltration and decreased goblet cells, while both MZD groups also had mild lymphocytic infiltration but increased goblet cells (Fig. 2e). More importantly, the intestinal mucosal layer in the untreated group was markedly attenuated relative to the healthy group, and this condition was effectively ameliorated by MZD intervention, resulting in substantial restoration of the mucosal thickness (Fig. 2f). No significant differences were found between the MZD-L and MZD-H groups in the intestinal propulsion rate, serum SOD and MDA levels, or mucosal thickness. The histopathological analysis revealed the impressive therapeutic efficacy of MZD therapy for age-related constipation treatment (Fig. 2g).

### MZD modulated gut microbiota diversity in aged constipated rats

**Figure 3:**
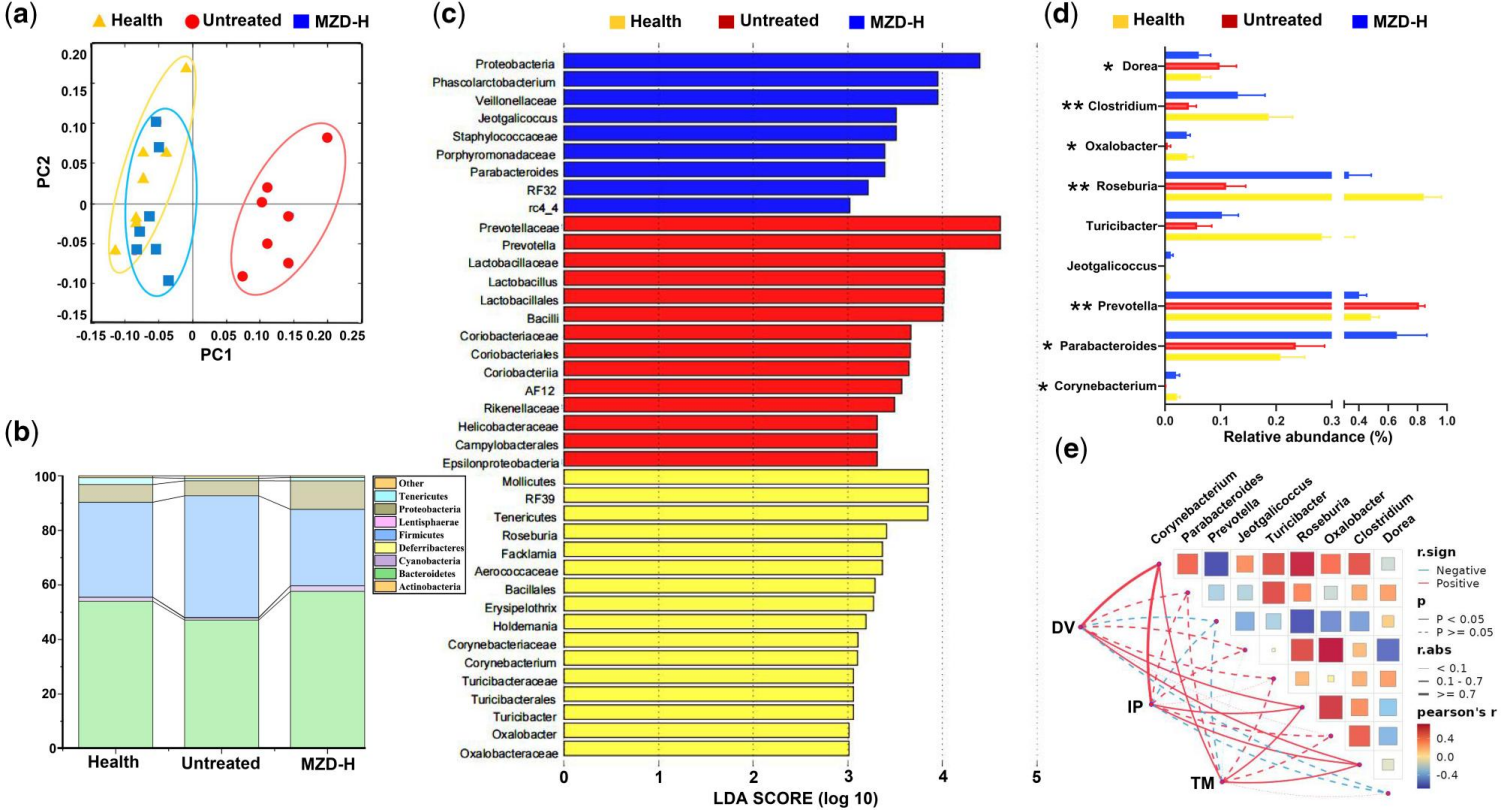
Structural comparison of fecal microbiota between the three groups. (a) PCoA plot generated using OTU metrics based on the Bray–Curtis similarity. (b) Bacterial taxonomic profiling on the phylum level of gut microbiota. (c) LDA coupled with effect-size measurements identified the most differentially abundant taxa. (d) Comparison of relative abundance at the genus level between the three groups. Values are expressed as mean ± SD. Student’s *t*-test *P* < 0.05, significant difference, *P < 0.05, **P < 0.01, untreated group vs MZD-H group. (e) Correlation analysis between constipation-related indexes and gut microbiota after MZD treatment. LDA = linear discriminant analysis, IP = ink-propelling rate, TM = thickness of the colon mucosa.

To determine the sample species composition and diversity, we classified the obtained effective sequences into OTUs. Supplementary Fig. S3 showed the OTU statistics of the three groups. As depicted in Supplementary Fig. S3, the healthy and MZD-H groups shared the highest number of common OTUs, whereas the healthy and untreated groups shared the lowest. According to Supplementary Fig. S4, the Chao1 index of the MZD-H group was higher than that of the untreated group, indicating that the MZD-H had significantly enhanced the microbial richness and diversity in the rats. Principal coordinates analysis (PCoA) results revealed significant clustering differences in the classification and function of colonic microorganisms between the healthy and MZD-H groups versus the untreated group, with each point corresponding to a single sample location (Fig. 3a). Thus, the MZD treatment had significantly increased the gut microbiota richness and diversity, and altered the microbial composition and function in rats compared with those in the untreated group.

The taxon summary revealed distinctive changes in the gut microbial composition in response to MZD administration. A total of eight phyla were detected in all samples, and the dominant phyla were Firmicutes, Bacteroidetes, Proteobacteria, and Tenericutes (Fig. 3b). The MZD-H group was similar to the control group. LEfSe was used to identify species with significant differences between groups. As shown in the cladogram, 39 microbial taxa were screened out by a linear discriminant analysis score of >3 (Fig. 3c). Changes in the microbiota composition after MZD administration were explored at the genus level (Fig. 3d). *Prevotella* and *Dorea* were significantly enriched in the untreated group. Previous studies have reported that *Prevotella* enhances susceptibility to chemical colitis, consistently with the pro-inflammatory potential of this organism [18]. *Dorea*, the main gas-producing bacterium in the intestines, is associated with increased intestinal permeability, which is thought to contribute to inflammatory bowel disease pathophysiology [19]. Specifically, the MZD-treated group exhibited a significantly higher abundance of beneficial bacteria, including *Corynebacterium* (increased by 6-fold), *Roseburia* (increased by 4-fold), and *Parabacteroides* and *Clostridium* (increased by 3-fold), all of which are prolific producers of short-chain fatty acids (SCFAs) [20–23]. We further analysed the correlation between changes in the gut microbiota and disease indicators. The abundance of *Corynebacterium*, *Roseburia*, and *Clostridium* was positively correlated with the DV, intestinal propulsion rate, and thickness of the colon mucosa in Fig. 3e.

### Fecal metabolic profiling after MZD treatment

**Figure 4:**
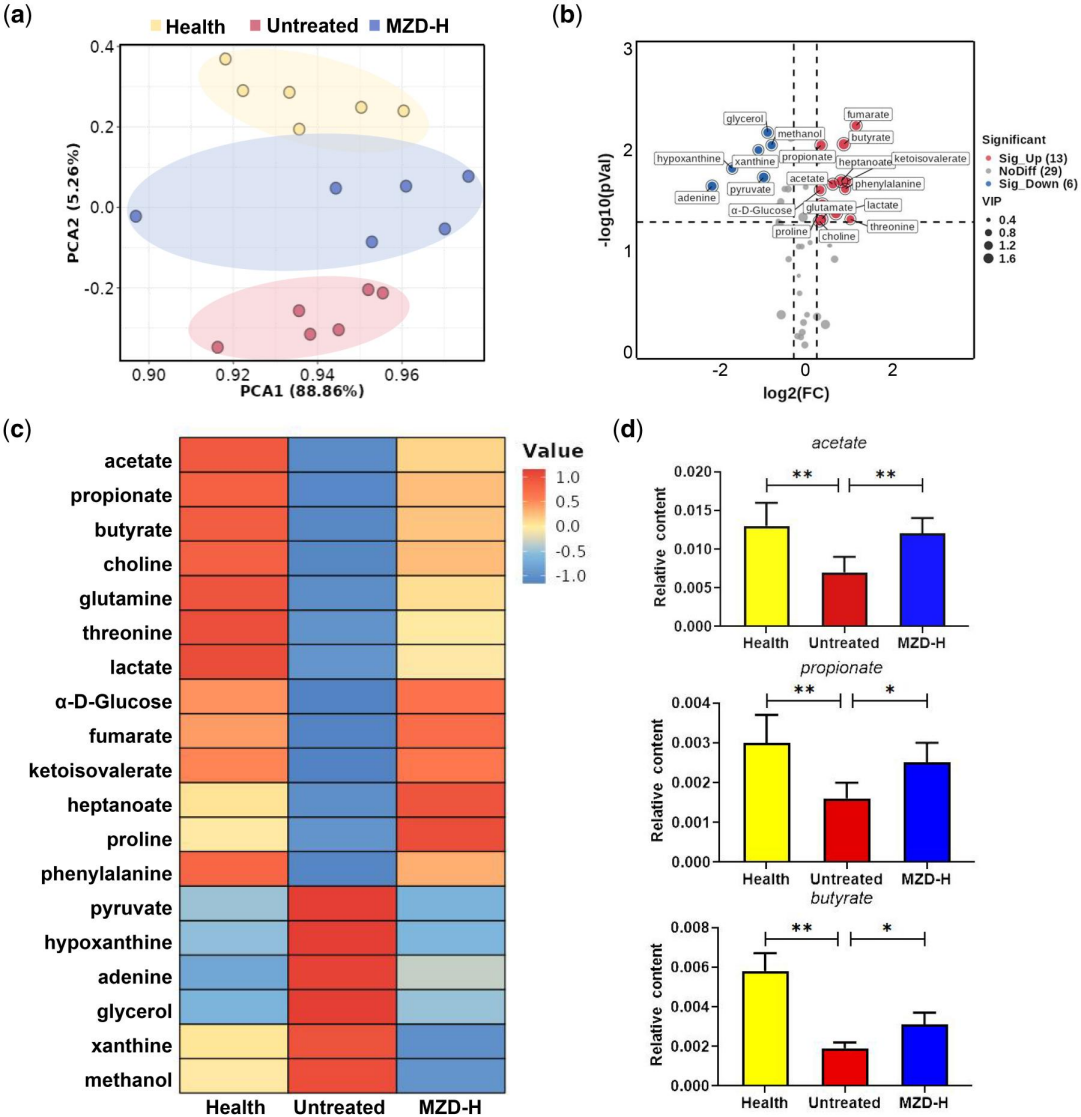
Significantly different fecal metabolites after MZD treatment. (a) PCA-score plots of feces metabolites for three groups. (b) Volcano plot of differential metabolites in the feces (untreated group vs MZD-H group; each dot represents a metabolite.) (c) Heat-map analysis of differential metabolites. (d) Relative contents of acetate, propionate, and butyrate. Values are expressed as mean ± SD. ANOVA *P* < 0.05, significant difference, *P < 0.05, **P < 0.01.

Multivariate analysis was performed by using principal component analysis (PCA) to reveal the clustering trends of each group. Each point in the PCA-score plot represents an individual sample and different samples were divided into blocks (Fig. 4a). Fig. 4a suggested that the untreated group showed a significantly different metabolic profile compared with the other two groups. A total of 48 metabolites were identified and the supervised pattern recognition of orthogonal partial least-squares discriminant analysis was used to screen out the differential metabolites present in the untreated group and the MZD-H group by using variable importance in the projection (VIP), as shown in Supplementary Fig. S5. On the basis of a VIP of >1, *t*-test *P* value of <0.05 (−log_10_  *P* value > 1.3), and a fold change (FC) of >1.2 or <0.83 (log_2_ FC > 0.263 or < −0.269), 19 metabolites were selected as the differential metabolites between the untreated group and the MZD-H group, and these results were listed in Supplementary Table S3. The variation tendencies of these metabolites were shown *via* a volcano plot in Fig. 4b. The results showed that 6 metabolites were significantly decreased in the MZD-H group, while 13 were significantly increased. The heat map showing the abundance of metabolites indicates that the MZD-H group was closer to the healthy group, suggesting that MZD may modulate metabolites altered during age-related constipation and help restore them to normal levels (Fig. 4c). It is considered that SCFAs are one of the significant metabolites of gut microbiota and we specifically showed the content of SCFAs in this study (Fig. 4d). As shown in Fig. 4d, MZD significantly increased the abundances of acetic acid, propionic acid, and butyric acid compared with those in the untreated group. Therefore, the effect of MZD on improving age-related constipation may be related to its regulation of gut microbiota metabolism, particularly the production of SCFAs.

### Effect of MZD on the colon in aged constipated rats

**Figure 5:**
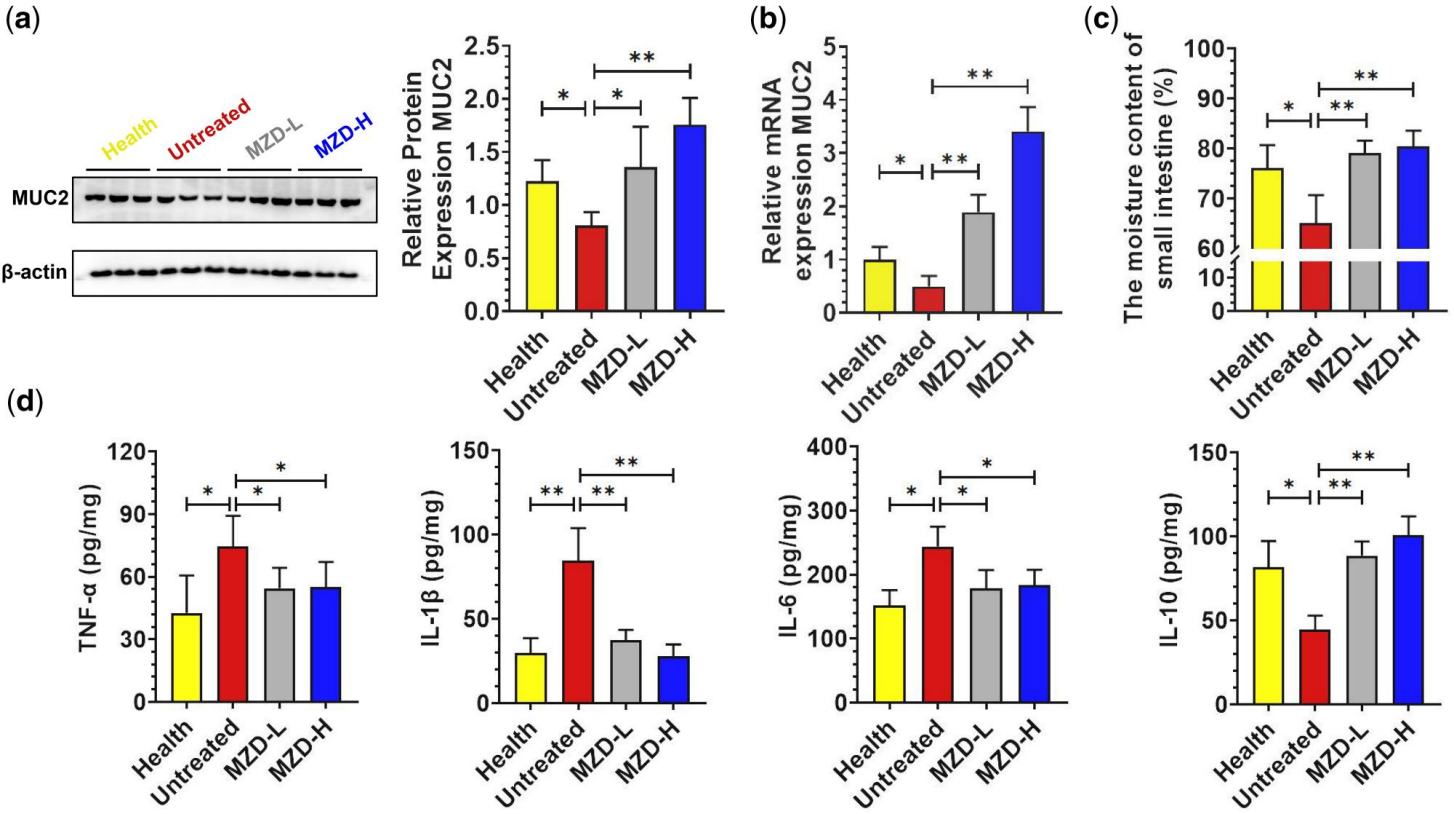
MZD promotes colon goblet-cell function in aged constipated rats. (a) Representative Western blot and densitometric analysis of MUC2. (b) Relative mRNA-expression level of MUC2. (c) Small-intestinal moisture content. (d) Colonic tissue levels of TNF-α, IL-1β, IL-6, and IL-10 measured by using ELISA. Values are expressed as mean ± SD. ANOVA *P* < 0.05, significant difference, *P < 0.05, **P < 0.01. TNF-α = tumor necrosis factor-alpha, IL-1β = interleukin-1 beta, IL-6 = interleukin-6, IL-10 = interleukin-10.

A decrease in intestinal mucus is responsible for constipation. Intestinal goblet cells are vital for protecting the intestinal epithelium by producing mucus. As shown in Fig. 2e, MZD treatment significantly increased the number of goblet cells, indicating that MZD can stimulate their proliferation. Mucin 2 (MUC2)—a key component of mucus—is secreted by goblet cells and forms a protective barrier to trap pathogens and toxins. The function of goblet cells and the expression of MUC2 are closely linked, together maintaining gut health [24]. Numerous studies have reported that SCFAs can stimulate the growth of goblet cells and promote their secretion of MUC2. Therefore, we further analysed the expression of MUC2 in the gut after MZD intervention. Western blot results showed that MUC2 expression was higher in the healthy and two MZD groups than in the untreated group (Fig. 5a). This conclusion was further validated by using quantitative real-time PCR (Fig. 5b). The mRNA expression levels of MUC2 were consistent with the protein expression levels detected by Western blot. Subsequently, the water content of the small intestines in the mice was measured. As expected, compared with the untreated group, MZD intervention significantly increased the moisture content of the small intestine (Fig. 5c). In addition, to determine whether MZD attenuates colonic inflammation, we measured the colonic levels of pro-inflammatory cytokines, including tumor necrosis factor-alpha (TNF-α), interleukin-1 beta (IL-1β), interleukin-6 (IL-6), and the anti-inflammatory cytokine interleukin-10 (IL-10), as shown in Fig. 5d. MZD markedly reduced TNF-α, IL-1β, and IL-6 while elevating IL-10 compared with the untreated group. MZD-L and MZD-H had similar effects, and there were no significant differences between the two groups.

**Figure 6:**
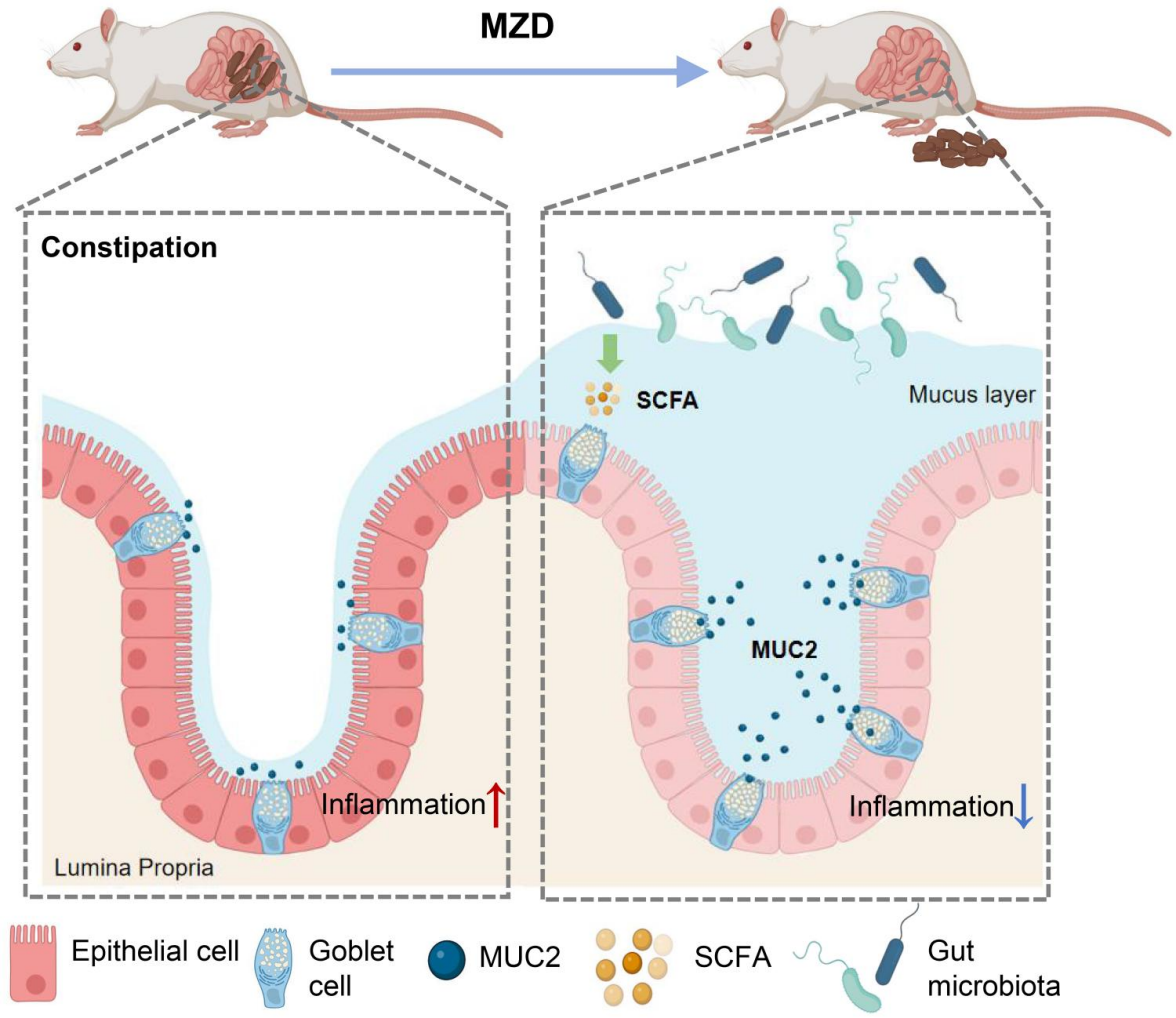
Schematic diagram showing the mechanism of the therapeutic effect of MZD on aged constipated rats. Created with Biorender.com (license number NY29BUKENH).

In summary, MZD alleviates age-related constipation by simultaneously dampening colonic inflammation and fortifying the mucus barrier. Figure 6 depicted the mechanistic workflow of MZD in alleviating aging-related constipation. The formula reshaped the gut microbiota to favor SCFA-producing taxa, raising local SCFA levels that stimulate goblet-cell proliferation and MUC2 secretion, and promoted the resolution of intestinal inflammation in aged constipated rats. Consequently, MZD orchestrated a mucus-secretory, barrier-reinforcing, and inflammation-resolving milieu that re-established epithelial homeostasis and accelerated fecal transit in aged constipated rats (Fig. 6).

## Discussion

Physiologically, constipation arises from two core defects: sluggish intestinal motility and a thinned, dehydrated mucus layer that hardens stool. Our data show that MZD counteracts both defects. The DV and ink-propulsion rate (a proxy for motility) both rose in aged constipated rats after MZD; this stimulant effect is attributable to the *Semen Raphani* and *Folium Sennae* [25, 26]. Simultaneously, MZD expanded the mucus layer by activating goblet cells—an action linked to *O. japonicus* [27]—and lowered oxidative stress by elevating SOD while suppressing MDA—an antioxidant benefit ascribed to *S. ningpoensis* and *A. membranaceus* [28, 29]. Thus, MZD integrates pro-motility, pro-mucus, and antioxidative actions to relieve constipation. However, the potential mechanism by which MZD treats age-related constipation remains not fully elucidated and requires further experimental validation.

Constipation can cause abdominal distension, belching, and gastric acid poisoning due to the failure of timely elimination of harmful substances produced in the body, resulting in intestinal ecological imbalance [30]. The gut microbiota changes significantly in the case of constipation, with the proportion of beneficial bacterial flora decreasing and the proportion of some neutral bacteria and harmful bacteria increasing [31, 32]. By modulating colonic motility, secretion, and absorption, gut microbiota may affect the development of constipation through microbial metabolic activities involving bile acids, SCFAs, 5-hydroxytryptamine, and methane [33]. Thus, the present study was designed to determine the effects of MZD treatment on fecal microbiota and microbiota-related metabolic phenotype alterations in aged constipated rats. However, the potential mechanism by which MZD treats age-related constipation remains not fully elucidated and requires further experimental validation.

SCFAs (acetate, propionate, and butyrate) serve as the principal energy currency of the colon, generated by the anaerobic fermentation of resistant starch and dietary fiber. Epidemiological evidence links adequate SCFAs to a reduced risk of colorectal cancer, colitis, and hypertension [33–35]. Acetate supplies ∼10% of human daily energy; propionate enters hepatic gluconeogenesis and may curb lipogenesis; butyrate fuels colonocytes, induces Treg cells, and enhances macrophage bactericidal activity, thereby attenuating colitis [36]. Critically, butyrate also upregulates MUC2 transcription and peptide secretion in goblet cells, thickening the protective mucus blanket [37]. Eight weeks of SCFA enemas improved mucosal healing in ulcerative colitis patients [38], whereas SCFA deficiency thins the barrier and raises endotoxin flux. Fig. 4 showed that MZD restored the fecal concentrations of acetate, propionate, and butyrate in aged constipated rats, re-establishing SCFA homeostasis. By normalizing these microbiota-derived metabolites, MZD simultaneously dampens inflammation and promotes mucin secretion, thereby meeting two key prerequisites for sustained relief from intestinal disorders.

Notably, the observed effects of MZD on constipation relief and SCFAs homeostasis may be mediated *via* modulation of the host–microbial metabolic axis, which is a directional hypothesis supported by the parallel changes in microbiota composition and metabolic phenotypes. However, the specific mechanisms underlying this axis modulation have not yet been experimentally demonstrated. To enhance the pharmacological reliability and mechanistic rigor of these findings, future studies will focus on benchmarking against standard control groups and verifying the causal mechanisms of the host–microbial metabolic axis, such as by incorporating standard positive controls to establish a reliable baseline for MZD-efficacy comparison and outcome reproducibility, and deploying microbe-manipulating mechanistic tools to verify the causal role of MZD on the host–microbial metabolic axis.

## Conclusions

In summary, our study investigated the therapeutic effects of MZD on age-related constipation in rats. MZD treatment alleviated age-related constipation by significantly enhancing intestinal peristalsis, increasing mucus secretion, and dampening inflammation through a multifaceted mechanism that includes modulating the gut microbiota, enhancing SCFA production, and upregulating MUC2 expression. Specifically, MZD enriched beneficial microbial taxa that produce SCFAs, which in turn suppresses pro-inflammatory cytokines, promoted goblet-cell proliferation, and stimulated mucus production by upregulating MUC2. Our findings highlight the therapeutic potential of MZD for age-related constipation and underscore the importance of the host–microbial metabolic axis in mediating its effects.

## Supplementary Material

goag031_Supplementary_Data
