# Supplementary material for: Effect of modified Zengye decoction on age-related constipation via modulation of the host–microbial metabolic axis
Source: Gastroenterol Rep (Oxf). 2026 May 13;14:goag031. doi: 10.1093/gastro/goag031 (PMC13176451; doi:10.1093/gastro/goag031)
Supplement: goag031_Supplementary_Data [file goag031_supplementary_data.docx]

Effect of modified Zengye decoction on age-related constipation *via* modulation of the host-microbial metabolic axis


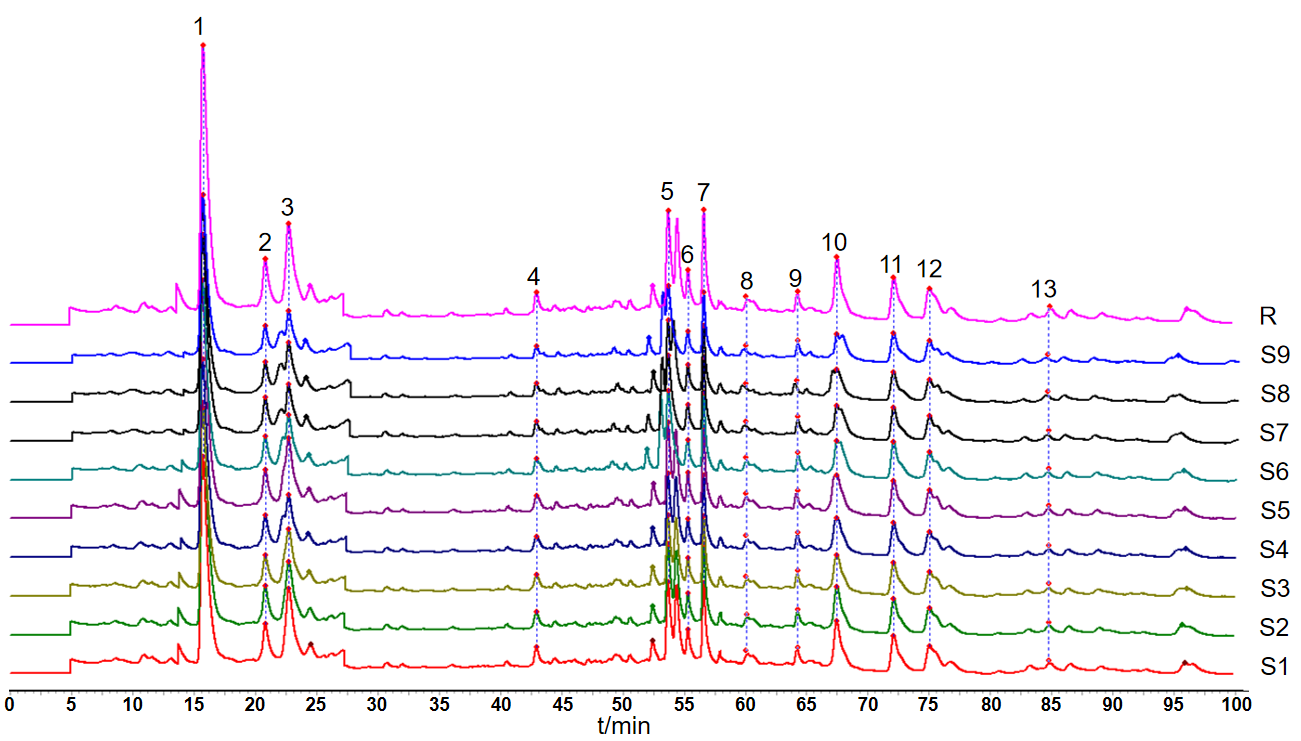


**Figure S1**. HPLC fingerprints of 9 batches of MZD and the reference fingerprint.

HPLC: high performance liquid chromatography; MZD: modified Zengye decoction.


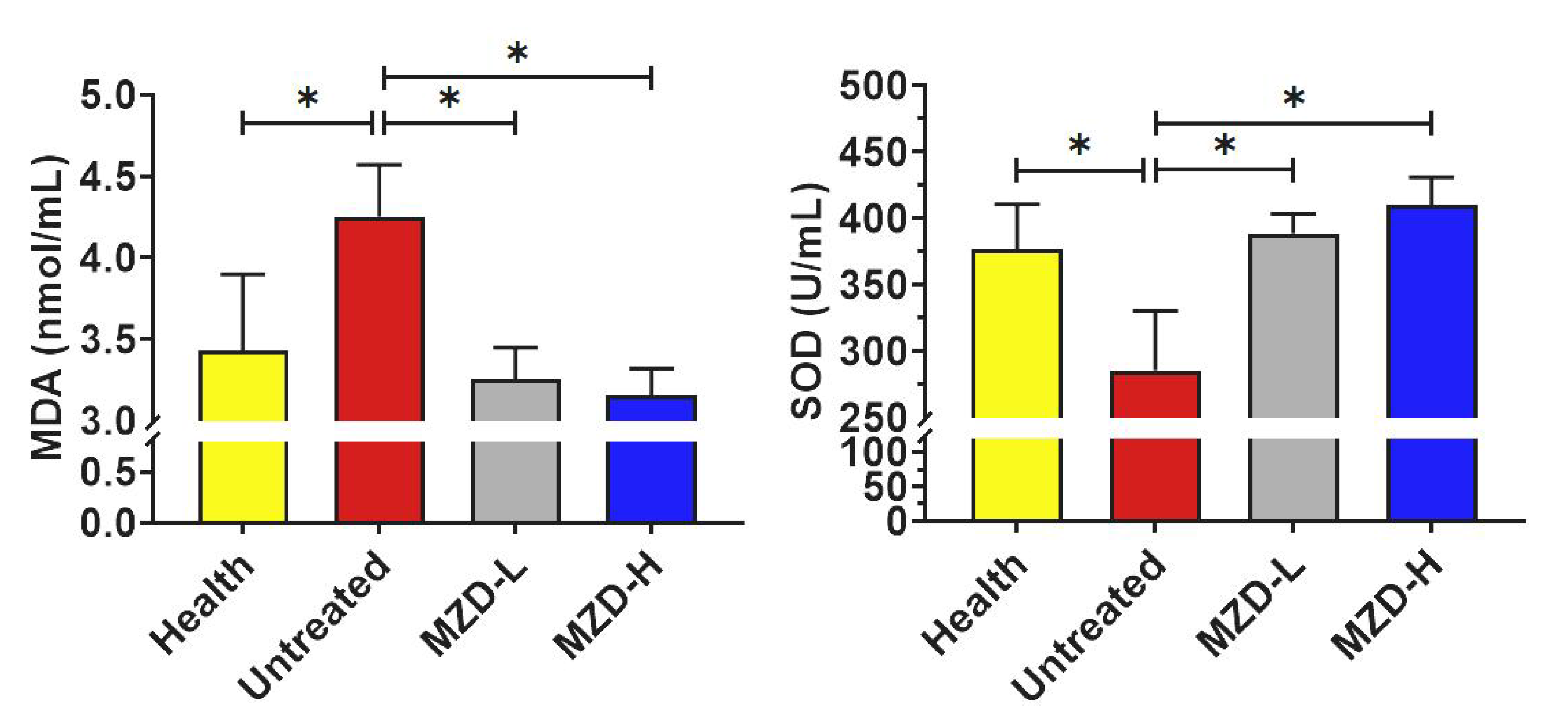


**Figure S2.** MDA and SOD levels in serum of rats after different treatments.

Values were expressed as Mean ± S.D. ANOVA *P* < 0.05, significant difference, * *P* < 0.05. MDA: malondialdehyde, SOD: superoxide dismutase, ANOVA: analysis of variance.


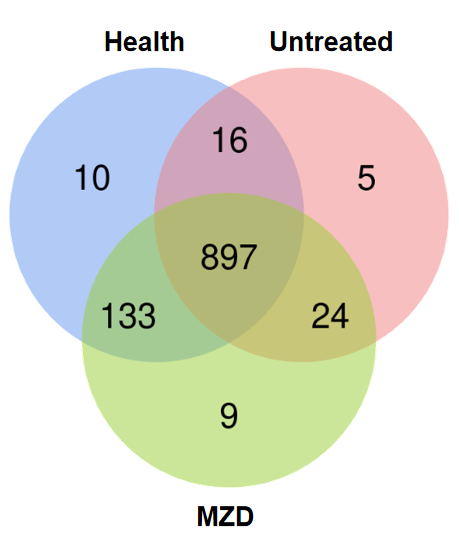


**Figure S3.** OTU Venn diagram of gut microbiota.

OTU: operational taxonomic unit.

**
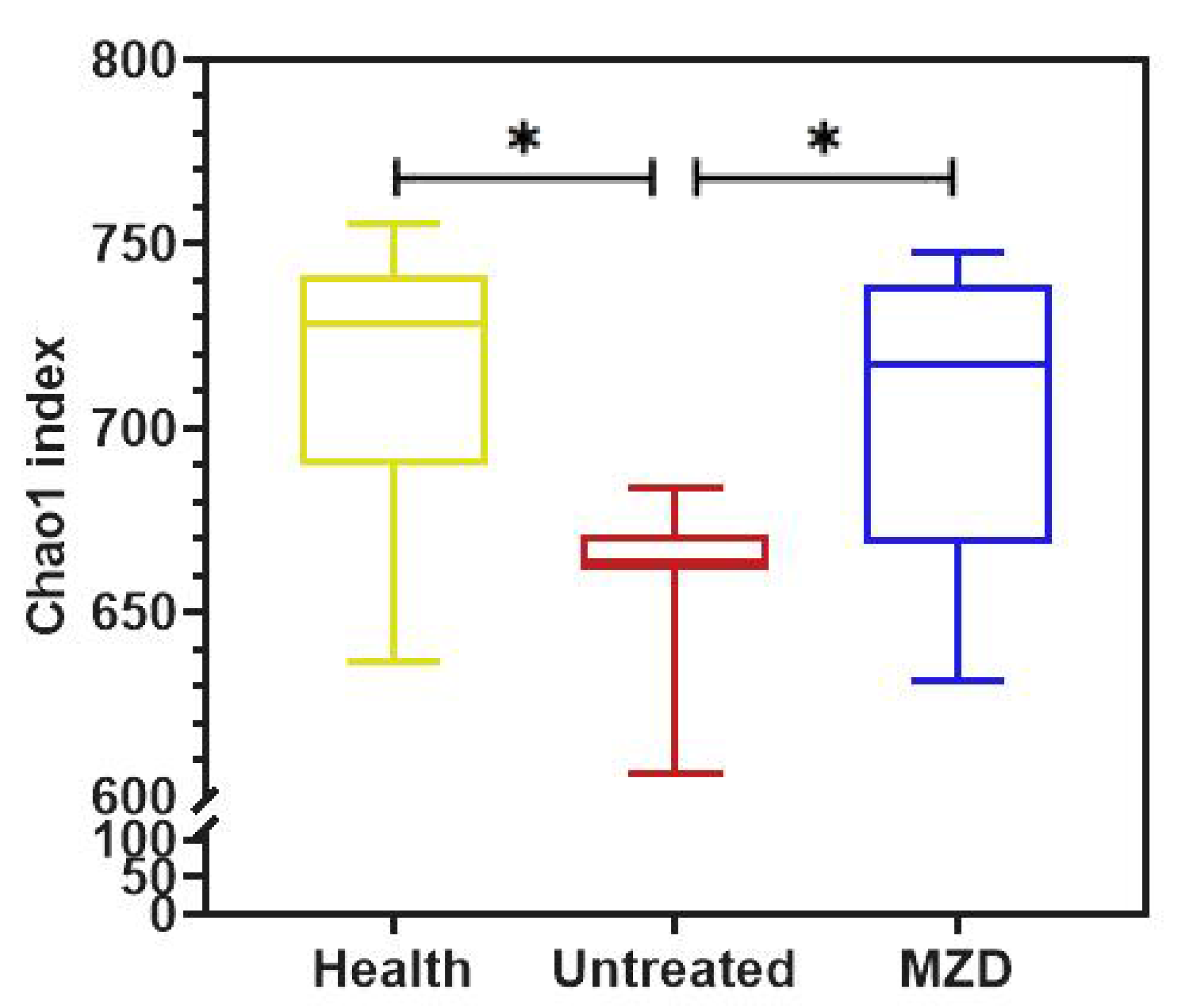
**

**Figure S4.** Chao1 index of gut microbiota in different groups. Chao1 index was used to estimate the total number of species in samples. larger Chao1 values indicate more species. ANOVA *P* < 0.05, significant difference, * *P* < 0.05.


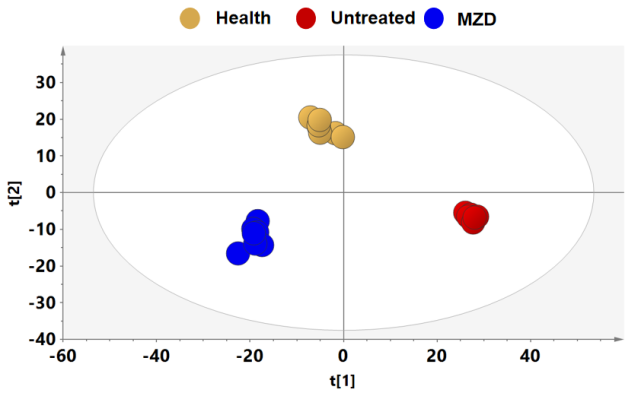


**Figure S5.** OPLS-DA score plots of the three groups in feces.

OPLS-DA: orthogonal partial least squares discriminant analysis.

**Table S1.** HPLC fingerprint similarity evaluation of the 9 batches MZD.

| NO. | S1 | S2 | S3 | S4 | S5 | S6 | S7 | S8 | S9 | R |
| --- | --- | --- | --- | --- | --- | --- | --- | --- | --- | --- |
| S1 | 1.000 | 0.996 | 0.999 | 0.999 | 0.999 | 0.997 | 0.997 | 0.996 | 0.992 | 0.999 |
| S2 | 0.996 | 1.000 | 0.993 | 0.995 | 0.994 | 0.991 | 0.993 | 0.990 | 0.995 | 0.998 |
| S3 | 0.999 | 0.993 | 1.000 | 0.999 | 0.999 | 0.998 | 0.995 | 0.994 | 0.992 | 0.996 |
| S4 | 0.999 | 0.995 | 0.999 | 1.000 | 0.998 | 0.996 | 0.997 | 0.997 | 0.992 | 0.998 |
| S5 | 0.999 | 0.994 | 0.999 | 0.998 | 1.000 | 0.997 | 0.995 | 0.996 | 0.992 | 0.998 |
| S6 | 0.997 | 0.991 | 0.998 | 0.996 | 0.997 | 1.000 | 0.993 | 0.991 | 0.992 | 0.995 |
| S7 | 0.997 | 0.993 | 0.995 | 0.997 | 0.995 | 0.993 | 1.000 | 0.997 | 0.995 | 0.996 |
| S8 | 0.996 | 0.990 | 0.994 | 0.997 | 0.996 | 0.991 | 0.997 | 1.000 | 0.994 | 0.994 |
| S9 | 0.992 | 0.995 | 0.992 | 0.992 | 0.992 | 0.992 | 0.995 | 0.994 | 1.000 | 0.999 |
| R | 0.999 | 0.998 | 0.996 | 0.998 | 0.998 | 0.995 | 0.996 | 0.994 | 0.999 | 1.000 |

**Table S2.** Sequences of amplificaton primers

| **Gene** | **Direction** | **Primer (5’→3’)** |
| --- | --- | --- |
| GAPDH | Forward | TGGCAAAGTGGAGATTGTTGCC |
|  | Reverse | AAGATGGTGATGGGCTTCCCG |
| MUC2 | Forward | GCTCAATCTCAGAAGGCGACAC |
|  | Reverse | CCAGATAACAATGATGCCAGAGC |

**Table S3.** Statistical analysis results of the metabolite change in feces

| **metabolites** | **δ 1H (ppm) and multiplicity** | **log2 FC** | **VIP** | **-log10 *P*** |
| --- | --- | --- | --- | --- |
| alanine | 1.48, 3.79 (d,q) | 0.04 | 0.66 | 0.35 |
| valine | 0.99, 1.04, 2.27, 3.62 (d,d,m,d) | -0.07 | 0.89 | 0.17 |
| N,N-dimethylglycine | 2.92, 3.71 (s,s) | 0.11 | 0.56 | 1.09 |
| butyrate* | 0.90, 1.56, 2.15 (t,m,t) | 0.88 | 1.87 | 2.10 |
| propionate* | 1.06, 2.19 (t,q) | 0.36 | 1.56 | 2.09 |
| lactate* | 1.33, 4.11 (d,q) | 0.70 | 1.76 | 1.39 |
| acetate* | 1.92 (s) | 0.63 | 1.45 | 1.69 |
| glutamate* | 2.10, 2.09, 2.36, 3.77 (m,m,m,m) | 0.38 | 1.45 | 1.33 |
| pyruvate* | 2.38 (s) | -0.95 | 1.61 | 1.76 |
| succinate | 2.41 (s) | -0.10 | 0.76 | 0.12 |
| U1 | 2.06 (s), | -0.04 | 0.87 | 0.27 |
| trimethylamine | 2.88 (s) | -0.08 | 0.67 | 1.05 |
| creatine | 3.04, 3.93 (s,s) | -0.15 | 0.67 | 1.05 |
| choline* | 3.21, 3.52, 4.07 (s,s,m) | 0.32 | 1.34 | 1.31 |
| methanol* | 3.37 (s) | -0.77 | 1.23 | 2.09 |
| β-galactose | 3.48, 3.65, 3.93, 3.71, 4.59, 3.74 (dd,dd,m,m,d,m) | -0.38 | 0.78 | 0.92 |
| β-D-Xylp-(1-4)d | 3.24, 3.58, 3.80, nd, nd, 4.58 (dd,t,m,d) | -0.15 | 0.45 | 0.75 |
| A2X2(Arabinoxylan)cα-L-Araf-(1-2)d | 4.13, 3.96, 4.14, nd, nd, 5.24 (dd,t,m,d) | -0.13 | 0.64 | 0.57 |
| uracil | 5.81, 7.54 (d,d) | 0.57 | 0.43 | 1.06 |
| fumarate* | 6.53 (s) | 1.16 | 1.45 | 2.29 |
| oligosaccharides | 3.92 (m) | -0.01 | 0.94 | 0.04 |
| β-glucose | 4.675, 3.235, 3.733 (d,d,dd) | -0.18 | 0.77 | 0.13 |
| heptanoate* | 1.309 (m) | 0.82 | 1.75 | 1.72 |
| methionine | 2.141, 2.169, 2.648, 3.853 (s,m,t,m) | -0.39 | 0.94 | 1.64 |
| sarcosine | 2.704, 3.597 (s,s) | -0.05 | 1.66 | 1.35 |
| malonate | 3.112 (s) | 0.15 | 0.34 | 1.26 |
| alpha-D-Glucose* | 3.425, 3.545, 3.835, 3.855, 5.242 (t,dd,m,dd,d) | 0.34 | 1.34 | 1.63 |
| glycine | 3.566 (s) | -0.62 | 0.66 | 1.47 |
| mcthylamine | 2.612 (s) | 0.01 | 0.67 | 1.66 |
| leucine | 0.959, 0.970, 1.696, 1.721, 1.738,3.740 (d,d,m,m,m,m) | 0.54 | 0.39 | 1.06 |
| aspartate | 2.683, 2.817, 3.903 (dd,dd,dd) | -0.59 | 1.34 | 1.28 |
| glycerol* | 3.56, 3.65, 3.77 (m,m,tt) | -0.86 | 1.34 | 2.22 |
| p-Hydroxyphenylacetate | 3.453, 6.867 ,7.183 (s,d,d) | 0.29 | 0.69 | 1.92 |
| hypoxanthine* | 8.197, 8.216 (s,s) | -1.67 | 1.23 | 1.85 |
| xanthine* | 7.920 (s) | -1.07 | 1.12 | 2.04 |
| N-Acetyl-D-glucosamine | 3.760, 3.872, 5.211 (m,m,d) | -0.62 | 0.56 | 1.31 |
| β-Ribofuranose | 4.005,5.258(m,d) | -0.47 | 0.77 | 1.64 |
| ketoisovalerate* | 1.13, 3.02(d,m) | 0.96 | 1.09 | 1.72 |
| taurine | 3.268, 3.425(d,t) | 0.67 | 0.99 | 0.92 |
| adenine* | 8.195, 8.213(s,s) | -2.13 | 1.45 | 1.67 |
| β-Mannose | 3.95, 4.93(m,d) | -0.55 | 1.56 | 0.35 |
| α-Arabinose | 3.82, 5.24(dd,d) | 0.46 | 1.55 | 0.25 |
| threonine* | 1.38, 3.59, 4.26(d,d,m) | 1.03 | 1.11 | 1.33 |
| N-Acetyl-glycoprotein | 2.03(s) | -0.33 | 0.93 | 2.16 |
| proline* | 2.01, 2.07, 2.36, 3.34, 3.45, 4.13(m,m,m,m,m,m) | 0.39 | 1.89 | 1.48 |
| tyrosine | 6.91, 7.20(d,d) | -0.56 | 1.05 | 1.51 |
| phenylalanine* | 7.33, 7.38, 7.43(m,m,m) | 0.91 | 1.19 | 1.64 |
| urocanate | 6.39, 7.34, 7.38, 7.90(d,d,s,s) | 0.27 | 1.23 | 0.33 |

FC: MZD group/untreated group; *: differential metabolites
